# *Schistosoma mansoni* Infection-Induced Transcriptional Changes in Hepatic Macrophage Metabolism Correlate With an Athero-Protective Phenotype

**DOI:** 10.3389/fimmu.2018.02580

**Published:** 2018-11-12

**Authors:** Diana Cortes-Selva, Andrew F. Elvington, Andrew Ready, Bartek Rajwa, Edward J. Pearce, Gwendalyn J. Randolph, Keke C. Fairfax

**Affiliations:** ^1^Department of Comparative Pathobiology, College of Veterinary Medicine, Purdue University, West Lafayette, IN, United States; ^2^Department of Pathology and Immunology, Washington University School of Medicine, St. Louis, MO, United States; ^3^Division of Health and Sport Sciences, Missouri Baptist University, St. Louis, MO, United States; ^4^Department of Basic Medical Sciences, Bindley Bioscience Center, College of Veterinary Medicine, Purdue University, West Lafayette, IN, United States; ^5^Department of Immunometabolism, Faculty of Biology, Max Planck Institute of Immunobiology and Epigenetics, University of Freiburg, Freiburg, Germany; ^6^Division of Microbiology and Immunology, Department of Pathology, University of Utah School of Medicine, Salt Lake City, UT, United States

**Keywords:** schistosomiasis, hepatic macrophages, metabolism, helminth, alternative activation of macrophages, atherosclerosis

## Abstract

Hepatic macrophages play an essential role in the granulomatous response to infection with the parasitic helminth *Schistosoma mansoni*, but the transcriptional changes that underlie this effect are poorly understood. To explore this, we sorted the two previously recognized hepatic macrophage populations (perivascular and Kupffer cells) from naïve and *S. mansoni*-infected male mice and performed microarray analysis as part of the Immunological Genome Project. The two hepatic macrophage populations exhibited remarkably different genomic profiles. However, this diversity was substantially reduced following infection with *S. mansoni*, and in fact, both populations demonstrated increases in transcripts of the monocyte lineage, suggesting that both populations may be replenished by monocytes following infection. Pathway analysis showed a profound alteration in global metabolic pathways, including changes to phospholipid and cholesterol metabolism, as well as amino acid biosynthesis and glucagon signaling. These changes suggest a possible mechanism for the previously reported athero-protective effects of *S. mansoni* infection. Indeed, we find that male ApoE null mice fed a high-fat diet in combination with *S. mansoni* infection have reduced plaque area and increased glucose tolerance as compared to control mice. Transcript analysis of infected and control high-fat diet fed ApoE^−/−^ mice confirm that *ApoC1, Psat1*, and *Gys1* are all altered by infection, suggesting that altered hepatic macrophage metabolism is associated with *S. mansoni*- induced protection from hyperlipidemia, atherosclerosis, and glucose intolerance. These results suggest a previously unknown and unreported role of hepatic macrophages in the modulation of whole body lipid and glucose metabolism during infection and provide a template for examining the role of immunomodulation on the long-term metabolism of the host.

## Introduction

Macrophages are highly plastic cells with vital functions in host defense and tissue repair and homeostasis (1). Their distinct functional characteristics depend on their distribution in different anatomical sites as well as the polarization signals from various tissue milieu (2–4). Due to their heterogeneity, a common classification of “classically activated” and “alternatively activated” has been attributed depending on their characteristic expression markers. While the classically activated macrophages (M1 macrophages) mediate autoimmunity and protection against different bacteria and viruses, alternatively activated macrophages (M2 macrophages) have been shown to play a role in wound healing, the regulation of inflammation, and metabolic functions (5, 6). Driven primarily by Toll-like receptor agonists and IFNγ, M1 polarized macrophages express inducible nitric oxide synthase (iNOS; *Nos2*) amongst other genes, and are pro-inflammatory, whereas M2 macrophages are induced by IL-4 and IL-13 and differentially express a range of genes including arginase-1 (*Arg1*), resistin like beta (Fizz-1; *Retnla*) and Ym1 (*Chil3*) (7). M2 macrophages have previously been shown to play a role in both the defense against some parasitic infections, as well as the transition to chronic helminth infection when immunomodulation and tissue repair is crucial for host survival (8). Importantly, they are the predominant macrophage in the liver during the Th2 dominated immune response to the helminth *Schistosoma mansoni*. In schistosomiasis, M2 macrophages are essential for survival of acute infection (9, 10) and provide proline, a collagen precursor, while also aiding in both the development and modulation of fibrosis and liver pathology (7). Recent studies have established that Arginase-1 producing M2 macrophages are essential for suppressing Th2 driven inflammation and fibrosis (11), and our recent work identified immune complex-driven production of IL-10 by these macrophages as critical for immunomodulation in chronic infection (12). However, knowledge about the transcriptional profile of distinct macrophage subsets during schistosome infection remains limited.

Mice infected with *S. mansoni* serve as a reproducible model for human schistosomiasis. In both humans and mice, the pathology progresses from an early Th1 response into a Th2 dominated one in response to egg deposition in organs such as liver, intestines, and lungs by worm pairs. Eggs are highly immunogenic and toxic, and the host response to this stimulus leads to fibrosis and portal hypertension (13). Previous studies have reported that schistosome infection in both humans and mice correlates with a significant modulation of lipid metabolism (14), lowering the total cholesterol levels of randomly bred mice and ApoE deficient mice (15), and reducing atherosclerotic lesions in ApoE deficient mice (16). Despite previous observations of the correlation of schistosome infection and lipid metabolic changes in both human and murine models, the mechanism by which *S. mansoni* exerts protection against cardiovascular disease is not understood.

Here we describe microarray data of the transcriptional profiles of hepatic macrophages in naïve and *S. mansoni* infected mice that indicate a reduction in the transcriptional heterogeneity of both Kupffer cells and perivascular macrophages following schistosome infection that suggests a shared function for these distinct macrophage populations. Surprisingly, a large percentage of the transcriptional changes observed in macrophages relate to genes involved in cholesterol and phospholipid metabolism, including ApoC1. These data suggest a pivotal role of hepatic macrophages in the modulation of lipid metabolism during infection. Furthermore, we confirm the anti-atherogenic effect of active schistosome infection in an ApoE^−/−^ atherosclerosis mouse model and postulate that this protection is due in part to significant reductions in serum ApoC1. Finally, we demonstrate that *S. mansoni* infection leads to a notable reduction in high fat diet (HFD) induced insulin resistance, and identify transcriptional changes to amino acid and glycogen metabolism that are likely mechanisms for this protection. Altogether, the data suggest an important and unexplored role of liver macrophages in the regulation of atherosclerosis and insulin sensitivity, a finding that has significant implications for diabetes, obesity and many other human diseases involving hyperlipidemia and insulin resistance.

## Materials and methods

### Ethics statement

This study was carried out in strict accordance with the recommendations in the Guide for the Care and Use of Laboratory Animals of the National Institutes of Health. The protocols were approved by the Institutional Animal Care and Use Committees of Washington University in St. Louis and Purdue University.

### Mice and parasites

Male C57BL/6J mice were purchased from Jackson Laboratories, *ApoE*^−/−^ (B6.129P2-Apoetm1Unc/J) mice were bred at Purdue University. Mice were kept under specific pathogen–free conditions and male mice were infected at 6 weeks of age. Snails infected with *S. mansoni* (strain NMRI, NR-21962) were provided by the Schistosome Research Reagent Resource Center for distribution by BEI Resources, NIAID NIH. Mice were exposed percutaneously to 75 *S. mansoni* cercariae. For experiments using *Apoe*^−/−^ mice, animals were transitioned to HFD (21% milk fat, 0.15% cholesterol; TD 88137 Harlan Teklad) 10 days prior to *S. mansoni* infection. Control mice were fed standard rodent chow (2018 rodent chow, Harlan Teklad) and mice were sacrificed at 10-weeks post-infection.

### Macrophage identification and isolation

Livers were removed from PBS-perfused animals, mashed, and incubated in RPMI (Mediatech) containing 250 μg/ml Collagenase D (Roche) at 37°C for 60 min. The resulting suspension was disrupted through a 100 μm metal cell strainer, washed, and then red blood cell lysed with ACK lysis buffer (BD) two times to remove hepatocytes followed by washing. The resulting pellet was washed and used for sorting. Surface staining with monoclonal antibodies, acquisition, and sorting were performed according to Immgen standard protocols (www.immgen.org). The following mAb (eBioscience, BioLegend, or R&D systems) against mouse antigens were used as PE, PE-Cy5, PE-Cy7, allophycocyanin (APC), APC-Cy7, Pacific blue, or biotin conjugates: CD11c (N418), CD11b (M1/70), CD45 (30-F11), MHC-II (M5/114.15.2), F4/80 (BM8), and MERTK (BAF591). Cells were directly sorted from mouse tissues and were processed from tissue procurement to a second round of sorting into TRizol within 4 h using a Beckton-Dickinson Aria II instrument.

### Microarray analysis, normalization, and dataset analysis

RNA was amplified and hybridized on the Affymetrix Mouse Gene 1.0 ST array by the Immgen consortium using double-sorted cell populations sorted directly into TRIzol. Data analysis utilized GenePattern analysis software. Raw data were normalized using the robust multi-array algorithm, returning linear values between 10 and 20,000. A common threshold for positive expression at 95% confidence across the dataset was determined to be 120. Differential gene expression signatures were identified and visualized using the “Multiplot” module of GenePattern (http://www.broadinstitute.org/cancer/software/genepattern/). Probesets were considered as differentially expressed with a coefficient of variation < 0.5 and a *p* < 0.05 (Student's *T*-test). Calculation and visualization of differential gene expression sizes (calculated as log_2_-transformed Hedges' g measures accompanied by 95% confidence intervals and *t*-test *p*-values indicating significance of differences, raw foldchanges, and log_2_-foldchanges) were performed using R-language for statistical computing.

To identify important genes (i.e., the genes expressed in a matter suggesting a link to macrophage class and/or infection status) we used various Boolean selection strategies focusing on multiple scenarios:

1. Genes which are upregulated in F4/80^high^ (Kupffer cells) (foldchange > 0), but downregulated in F4/80^int^ (perivascular macrophages) (foldchange < 0).
2. Genes which are downregulated in F4/80^high^ (foldchange < 0), but upregulated in F4/80^int^ (foldchange > 0)
3. Genes which are significantly (Benjamini-Hochberg adjusted p < 0.05) upregulated in both cell types
4. Genes which are significantly (Benjamini-Hochberg adjusted p < 0.05) downregulated in both cell type
5. Genes which differ substantially in the degree of regulation (regardless of the direction of regulation).

The described Boolean strategies are illustrated in the Venn diagrams in Figures **2A–C**. The following set designations were used in the set notation:

- q_hi = Significantly regulated genes in F4/80^high^ cells (BH adjusted *p* < 0.05)
- q_nt = Significantly regulated genes in F4/80^int^ cells (BH adjusted *p* < 0.05)
- hi_up = Genes upregulated in F4/80^high^ cells
- int_up = Genes upregulated genes in F4/80^int^ cells
- hi_down = Genes downregulated in F4/80^high^ cells
- int_down = Genes downregulated in F4/80^int^ cells

The employed set notation:

- A∩B–A intersect B
- A∪B–A union B
- A\B–A minus B, or A complement B

The Data (significantly impacted pathways, biological processes, molecular interactions.) were analyzed using Advaita Bio's iPathwayGuide (http://www.advaitabio.com/ipathwayguide). Pathway analysis was performed on log_2_-transformed data using Bonferroni-corrected *p*-values. Data are deposited in GenBank under accession no. GSE37448 as part of the Immgen 2 dataset.

### Principle component analysis

For unsupervised visualization of the gene expression profiles via principal component analysis, the gene expression data were constrained to a simplex and transformed using centered log-ratio transformation. The resultant values were ordered according to their variances, and the top 0.5-percentile of the genes were selected for the computation of the principal components (PCs).

### Flow cytometric analysis

Livers were perfused with 1X PBS, mashed and digested in DMEM containing 250 μg/ml Collagenase (Sigma) at 37°C for 30 min. The digested livers were then mashed and filtered through a 100 μm metal strainer and digestion was repeated for 15 min. Total liver contents were strained and washed with DMEM. The pellet was lysed with 1X lysis buffer (BD PharmLyse), quenched, and washed. The resulting cell suspension was used in flow cytometry. Surface staining was performed using the following mAb against mouse antigens: CD45 (30-F11, eBioscience), CD301(BioRad), CD206 (C068C2, Biolegend), F4/80 (BM8, Biolegend), mouse Mer biotinylated (R&D), CD64(X54-5/7.1, BD). Samples were acquired using FACSCanto II flow cytometer (BD) and analyzed using Flowjo X 10.0.7r2 (FlowJo LLC, Inc.,).

### Analysis of atherosclerotic plaques

At sacrifice, mice were perfused with PBS, and the heart was extracted for plaque assessment at the aortic sinus. Following fixation in 4% paraformaldehyde, tissue was cryoprotected in 30% sucrose, embedded in OCT compound (Fisher Scientific), and flash frozen. Fifteen-micron cryosections were made in the aortic sinus from initiation to termination of the aortic valve leaflets. Plaque assessment in the aortic sinus was performed as previously described (17), and is briefly described as follows. The total plaque was determined by taking the average of total atherosclerotic plaque from at least five sections at least 60 microns apart. Macrophage content was determined using CD68 immunofluorescence: average CD68 (BioRad) positive area was assessed from at least five sections at least 60 microns apart. Quantification of plaque area and area occupied by CD68 was calculated with ImageJ software.

### Cholesterol quantitation

Total cholesterol was measured from plasma as previously described (17) using colorimetric assessment methods. In brief, whole blood was collected by mandibular bleed into EDTA-plasma collection tubes, and the plasma fraction was removed following red blood cell sedimentation via centrifugation. Plasma samples were stored at −80°C until use. Total plasma cholesterol was assessed by manufacturer's protocol (Wako Diagnostics, #999-02601). Values were calculated from the mean of two replicates from a standard calibration curve. Total plasma cholesterol was reported as mg per dL.

### RNA isolation and q-RT-PCR analysis

Sections of perfused livers were homogenized in Trizol, and RNA isolation was performed as previously described [Immunological Genome Project, Total RNA isolation with Trizol; (18)]. RNA was used for cDNA synthesis using Superscript II (Invitrogen) for qPCR analysis. qPCR was performed using TaqMan Gene expression assays (gys1, CD36, ApoC1, ThermoFisher) or SYBR Green [psat1 (19), primers forward: ACG CCA AAG GAG ACG AAG CT and reverse: ATG TTG AGT TCT ACC GCC TTG TC] on an Applied Biosystems Stepone Plus Real-Time PCR System. Beta-Actin assay number Mm00607939_s1, GYS1 assay Mm01962575_s1, CD36 assay Mm00432403_m1, ApoC1 assay Mm00431816_m1. Relative expression was calculated using the 2-^ΔΔ*Ct*^ method.

### ELISA

ApoC1 plasma concentrations were determined by ELISA using an anti-mouse ApoC1 ELISA kit as per the manufacturer's instructions (LSBio).

### Statistics

Statistical analyses for non-microarray data were performed using one-way ANOVA, a non-parametric Mann-Whitney test, or unpaired Student's t-test depending on the distribution of the data. *P* ≤ 0.05 were considered statistically significant. Graph generation and statistical analyses were performed using Prism (GraphPad v7.0) and R-language for statistical computing.

## Results

### *S. mansoni* infection profoundly alters hepatic macrophage metabolism

**Figure 1:**
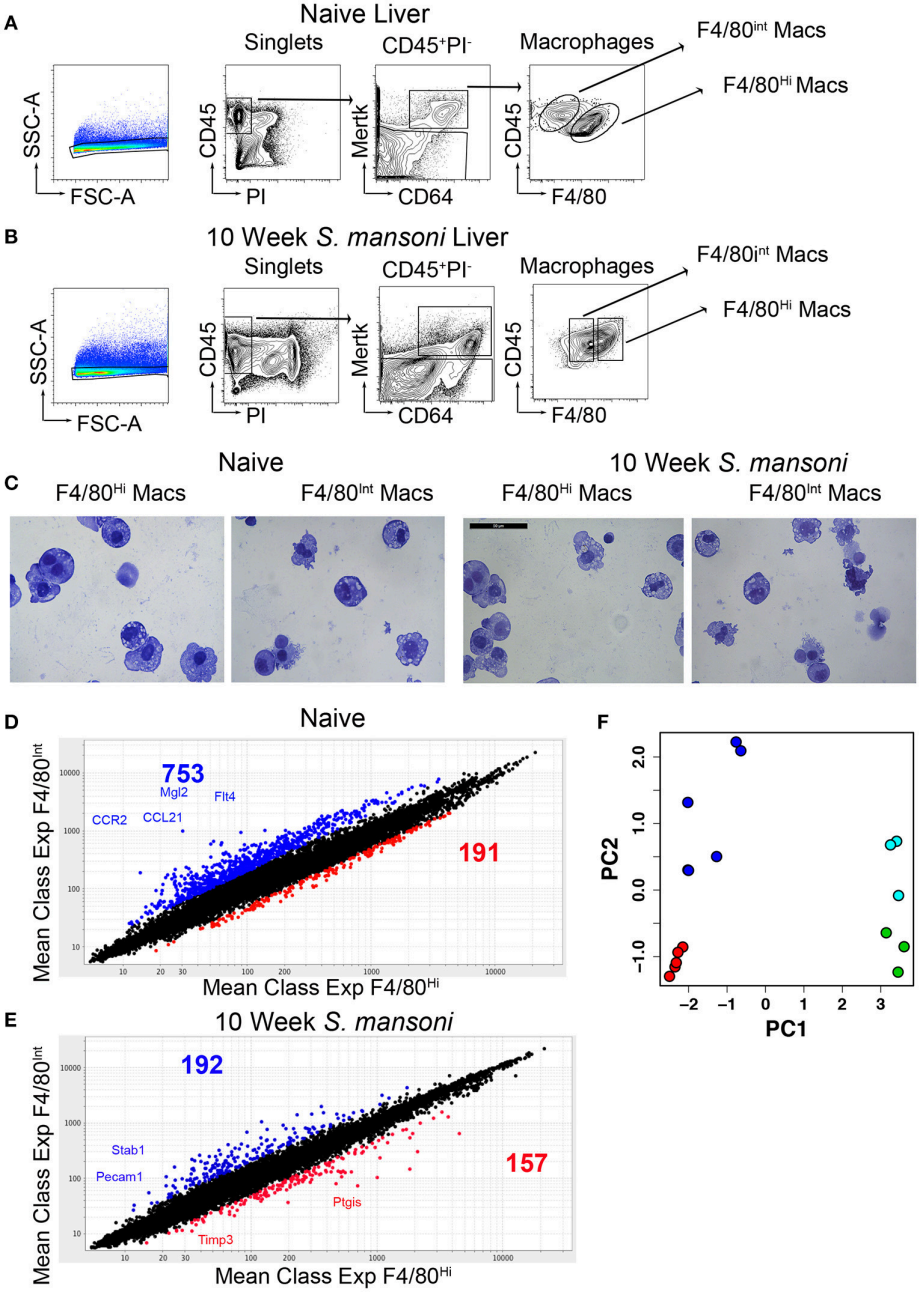
Murine livers contain two distinct macrophage populations and *S. mansoni* infection decrease transcript diversity between Kupffer cells and perivascular macrophages. C57BL/6 male mice were either naïve or infected with *S. mansoni* for the indicated number of weeks. **(A,B)** Perfused livers were removed, digested with collagenase for 1 h, and disrupted through a metal strainer to create single cell suspensions. Cells were identified by staining for the indicated markers and sorted on an Aria II. All populations were sorted two times, with the second sort going directly into Trizol according to the Immgen protocol. Individual animals were sorted from multiple litters/infections to achieve 4-5 biological replicates for individual microarray analysis. **(C)** Representative cytospins of aliquots from the second sorts of each macrophage populations were stained with H&E. **(D,E)** Total RNA was extracted, amplified, and transcript expression determined using whole-mouse genome Affymetrix Mouse Gene 2.0 ST Arrays through the Immunological genome consortium. **(D)** The number of probes increased by 2-fold with a *p* > 0.05 Blue indicates increased expression in F4/80^int^ liver macrophages at steady state, Red indicates increased expression in F4/80^hi^ liver macrophages at steady state. **(E)** The number of probes increased by 2-fold with a *p* > 0.05 Blue indicates increased expression in F4/80^int^ liver macrophages at steady state, Red indicates increased expression in F4/80^hi^ liver macrophages at 10-weeks post *S. mansoni* infection. Each group contained 3-5 individual RNA samples. **(F)** Principal component plot showing differences in gene expression profiles between samples of naïve F4/80^high^ cells (green), 10-week *S. mansoni* F4/80^high^ cells (red), naïve F4/80^int^ cells (cyan), and 10-week *S. mansoni* F4/80^int^ cells (blue).

While it is widely accepted that hepatic macrophages play a critical role in the pathology and ultimately the survival of *S. mansoni* infection, the transcriptional changes that accompany the response to *S. mansoni* antigens are not entirely understood. We have previously established 10-weeks post-infection as a key time-point in immunomodulation where SEA (schistosome egg antigen) specific B cells are recruited to the liver, and immune-complex ligation of hepatic macrophages begins, leading to macrophage IL-10 production (12). To explore the transcriptional changes to liver macrophages associated with *S. mansoni* infection, we first identified hepatic macrophages (CD45^+^PI^−^Mertk^+^CD64^+^) and then sorted F4/80 ^high^ (Kupffer cells) and F4/80^int^ (perivascular macrophages) populations from the livers of naïve and 10-week old *S. mansoni*-infected C57BL/6 mice according to Immgen standard protocols (4, 20), Figures 1A,B]. Cytospin examination of cells from both naïve and infected mice confirmed that both populations were macrophages, with the morphology of F4/80 ^high^ macrophages displaying greater size heterogeneity than the F4/80^int^ macrophages (Figure 1C).

We compared the gene expression profile of naïve Kupffer cells and perivascular macrophages and found very high transcriptional diversity between these two macrophage populations considering that they reside in the same tissue environment (Figure 1D). Subsequently, we performed the same comparison for 10-week post-infection samples and observed that transcript diversity decreases following *S. mansoni* infection (Figure 1E), suggesting that Kupffer cells and perivascular macrophages may have shared functions in response to infection. Additionally, some of the transcripts that increased in both populations following infection (i.e., Ly6C) were consistent with both macrophage populations being repopulated by monocytes at this time-point, as recently described (21, 22). A recent report (23) indicated that in models of sterile liver injury, peritoneal macrophages are recruited to the liver to aid in tissue repair, we did not, however, detect increases in Gata6 transcripts in our microarrays, so this paradigm may not apply to *S. mansoni* infection. We then performed an unsupervised visualization of the gene expression profiles before and after *S. mansoni* infection via principal component analysis (Figure 1F). The plot demonstrated that the PC 1 is associated with the difference between cells from the naïve and the 10-week *S. mansoni*-infected animals, whereas the PC 2 described the difference between the F4/80^high^ and the F4/80^int^ cells regardless of the infection status. The ten genes with the highest contribution to PC 1 are Slc7a2, Timd4, Flrt2, F7, Atp6v0d2, Pdcd1lg2, Arg1, Ch25h, ApoC1, and Dhrs9. The 10 genes with the highest contribution to PC 2 are Lum, Lox, Dpt, Cd163, Slc7a11, Igk-V1, Mgp, Jam2, Emcn, and Arhgap29. The effect sizes of the differences in the gene expressions represented as two-dimensional Mahalanobis distances in PC space are 18.83 (naïve F4/80^int^ cells vs. 10-week *S. mansoni* F4/80^int^ cells), 35.32 (naïve F4/80^high^ cells vs. 10-week *S. mansoni* F4/80^high^ cells). In both cases, the naïve vs. infected differences were statistically significant (Hoteling T^2^ test, *p* < 10^−5^). Although distances for naïve F4/80^high^ vs. naïve F4/80^int^ cells and 10 -week infected F4/80^high^ vs. 10-week-infected F4/80^low^ cells were similar (4.40 and 4.09, respectively), the larger variance among naïve F4/80^int^ cells made the former pair of expressions indistinguishable in the PC space (Hoteling T^2^ test, p>0.1). On the other hand, the dissimilarity between the gene expressions identified in the cells from the infected animals was significant (*p* = 0.004).

**Figure 2:**
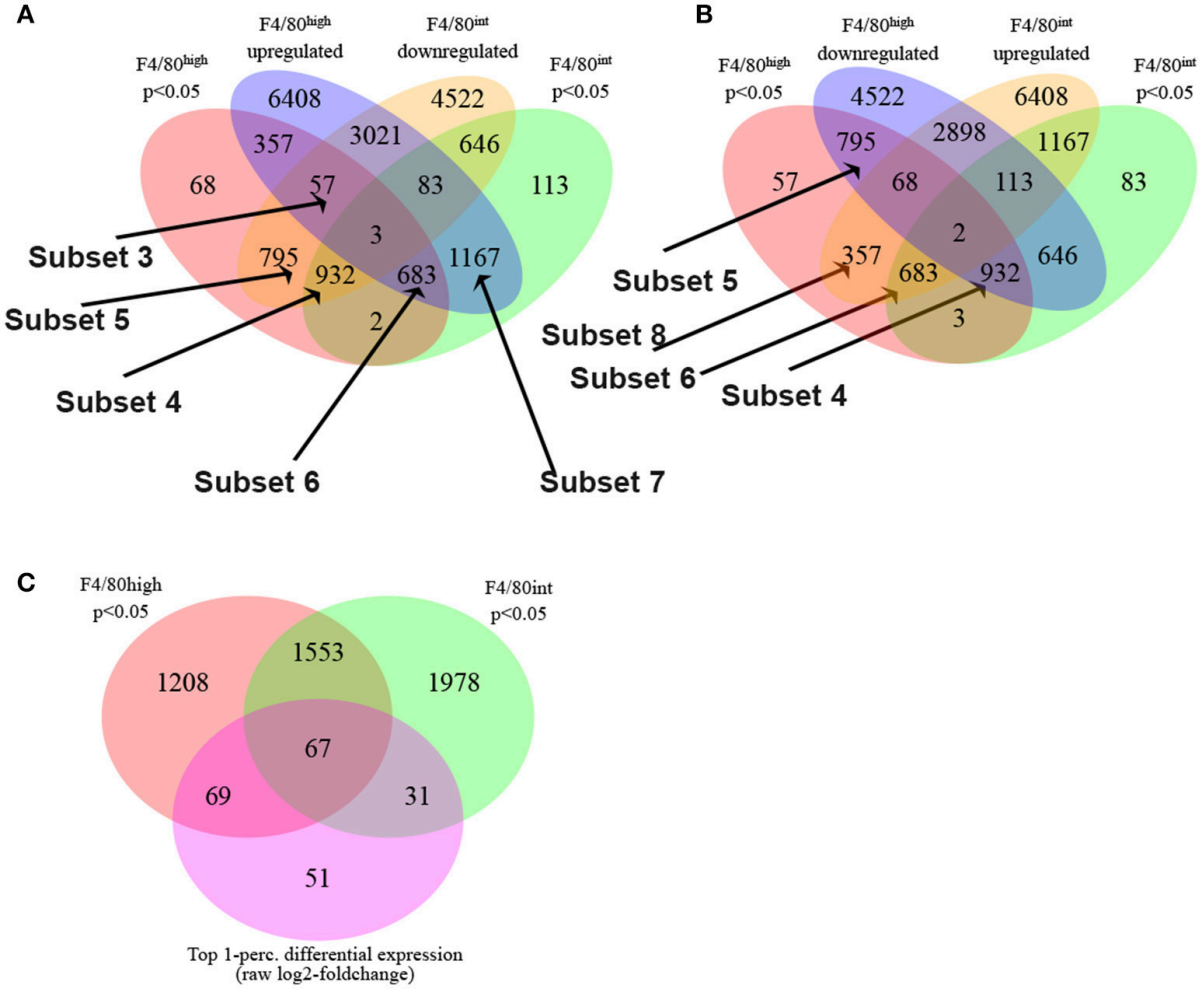
*S. mansoni* infection induces profound transcriptional alterations to Kupffer cells and Perivascular macrophages. **(A,B)** The Boolean strategies described in the methods are illustrated in the Venn diagrams: The following set designations were used in the set notation:
q_hi = Significantly regulated genes in F4/80^high^ cells (BH adjusted p < 0.05)q_nt = Significantly regulated genes in F4/80^int^ cells (BH adjusted p < 0.05)hi_up = Genes upregulated in F4/80^high^ cellsint_up = Genes upregulated genes in F4/80^int^ cellshi_down = Genes downregulated in F4/80^high^ cellsint_down = Genes downregulated in F4/80^int^ cells The employed set notation:
A∩B–A intersect BA∪B–A union BA\B–A minus B, or A complement B **(C)** Top 1-perc. differential expression (normalized log2-foldchange) in perivascular and Kupffer cells.

Next, we employed the Boolean selection strategies detailed in the methods section to compare the transcriptional profiles of Kupffer cells and perivascular macrophages from 10-week infected animals to their naïve counterparts, and identified numerous subsets of differentially regulated genes (Figure 2). Subsets 3, 4, and 6 shed light on the putative functional roles of these two cell types in response to *S. mansoni* infection. Among 57 genes in the Subset 3 (q_hi⋂hi_up⋂int_down)_(q_int), we located Cbr2 (carbonyl reductase 2, involved in arachidonic acid metabolism), which is significantly (*p* < 0.05) and highly (fold size = 20.7) upregulated in F4/80^high^, but does not demonstrate a statistically significant change in F4/80^int^, Alox (*p* < 0.05, fold size = 5.09), and F13a1 (*p* < 0.05, fold size 20). Additionally, among 932 genes in Subset 4 (q_hi⋂q_int⋂int_down)_(hi_up), which is equivalent to (q_hi⋂q_int⋂int_down⋂hi_down) and contains the genes which are significantly downregulated (foldsize < 0) in both cell types we found Adh1, Alb, Aldh2, Apoa2, ApoB, ApoC1, ApoC3, Cd55, Clec4f, Creg1, Fabp7, Flt4, Gpr116, Hmcn1, Hpgd, Marco, Pde7b, Ptprb, Slc16a9, Slc40a1, Slco2a1, St3gal5, and Stab2. Surprisingly, this subset contains many genes involved in phospholipid and cholesterol metabolism, as well as solute transport. Further, we found that Prg4 and Spic belong to a Subset 5 of 795 genes that are downregulated in both macrophage types, but the downregulation in F4/80^int^ has not been statistically significant. The set is designated as (q_hi⋂int_down)_(q_int∪hi_up). 683 genes were significantly simultaneously upregulated in F4/80^int^ and F4/80^high^ forming a Subset 6 (Figure 2A), (q_hi⋂q_int⋂hi_up)_(int_down) = (q_hi⋂q_int⋂hi_up⋂int_up). Among them we identified Anxa1, Arg,1 Atp1a3, Ccr2, Cd63, Chi3l3, Chi3l4, Dhrs9, Ear11, Egr2, Fcrlb, Fn1, Gda, Mgl2, Nfil3, Nos2, Pdcd1lg2, Retnla, Slc41a2, Slc7a2, and Uck2. This set contains many of the genes that are known to be hallmarks of alternative activation. Our filtering strategy identified 1167 genes belonging to (q_int⋂hi_up)_(q_hi∪int_down), denoted as Subset 7. These genes demonstrate upregulation in both populations of macrophages; however, the upregulation in F4/80^high^ is not statistically significant. Among them, we found Ccl2, Ccl7, and Ptgs2. Another interesting subset of 357 genes (q_hi⋂hi_up)_(q_int∪int_down) contained upregulated genes, which changed in a statistically significant manner in F4/80^high^ but not in F4/80^int^. Ccl12 with a foldchange of ~15 and Ccl8 with a very high foldchange of 76.6 in F4/80^high^ as well as Mfsd6 were among these genes. Interestingly, there were only three genes in subset 1 (q_hi⋂q_int⋂hi_up⋂int_down): Atrnl1, Mef2c, Nav1, and only 2 genes in subset 2 (q_hi⋂q_int)_(hi_up∪int_down): Igf2bp2 and Ube2j2 (Figure 2A), none of these genes had a particularly high absolute fold-change, further suggesting that both perivascular macrophages and Kupffer cells have a shared response to *S. mansoni* infection.

Some genes which were upregulated in both types of macrophages (or downregulated in both) nevertheless showed substantial differences in the level of regulation, exhibited by a large disparity in foldchanges. For instance, Rnase4 was upregulated in F4/80^high^ and F4/80^int^; however, in the first case the observed foldchange was 11, and in the second instance is only 1.6. To further delineate key differential transcriptional changes, we used the absolute difference between log_2_-foldchanges as a direct metric of differential regulation. The top 1-percentile of genes sorted by this measure contains 67 transcripts (Figure 2C) expressed with log_2_-foldchange difference ranging from 2.75 to 1.4. Among them, there are some of the known hallmarks of alternative activation (in the order of differences) Mgl2 (F4/80^high^ foldchange = 35.8, F4/80^int^ foldchange = 7.5), Retnla (F4/80^high^ foldchange = 84.2, F4/80^int^ foldchange = 19.5) as well as Ear11, Chi3l3, Ptprb, Ccr2, Gda, Gpr116, St3gal5, Stab2, Cd63.

**Figure 3:**
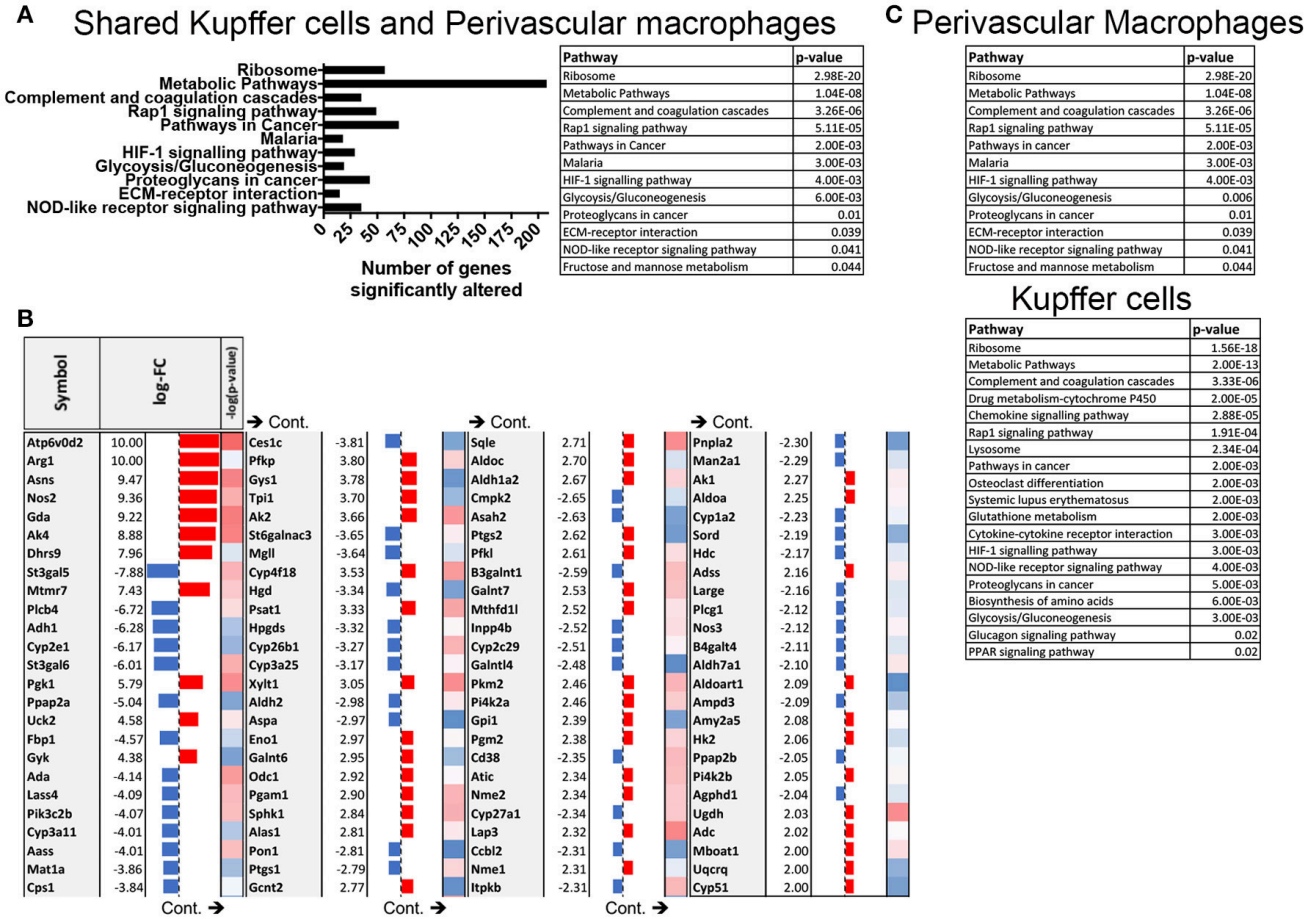
*S. mansoni* infection induces profound alterations to metabolic pathways in hepatic macrophages. Expression data from microarrays were log transformed and *p*-values were calculated in R. Corrected *P*-values and fold changes were used for pathway analysis. **(A)** Pathways that are significantly altered in both perivascular macrophages and Kupffer cells at 10-weeks post *S. mansoni* infection as calculated using Advaita Corporation iPathwayGuide table depicts pathway name and *p*-values are Bonferroni corrected **(B)** The table shows top absolute values of the log-fold changes in both perivascular macrophages and Kupffer cells at 10 weeks post *S. mansoni* infection (all log-FC≥2) of genes identified in the KEGG metabolic pathway using Advaita Corporation iPathwayGuide. The –log_10_ of the *p*-values range from 1.3 (blue) to 3.5 (red). **(C)** Tables listing pathway name and Bonferroni corrected *p*-value of the significantly altered pathways in Perivascular macrophages and kupffer cells as calculated using Advaita Corporation iPathwayGuide.

These data prompted us to conduct pathway analysis using the Advaita iPathwayGuide to uncover the pathways that are significantly manipulated by *S. mansoni* infection. Meta-analysis of transcriptional changes shared across both hepatic macrophage populations revealed striking alterations to multiple KEGG pathways, including multiple pathways of inflammation and cellular metabolism (Figure 3A), with the KEGG metabolic pathway showing the highest number of altered genes (Figures 3A,B, pathway Bonferroni corrected *p* = 1.042 × 10^−8^ and Supplementary Figure 1). While some of these metabolic genes have previously been linked to *S. mansoni* infection (i.e., Arg1 and Nos2), others were not previously considered to be associated with either alternative activation, or *S. mansoni* infection (i.e., Gys1, Psat1). We conclude that *S. mansoni* infection strongly alters the metabolic potential of liver macrophages. Since the liver is responsible for regulating many aspects of whole body metabolism (24, 25), these alterations may have profound effects on infected individuals.

### Infection reduces ApoC1 production and aortic plaque formation

**Figure 4:**
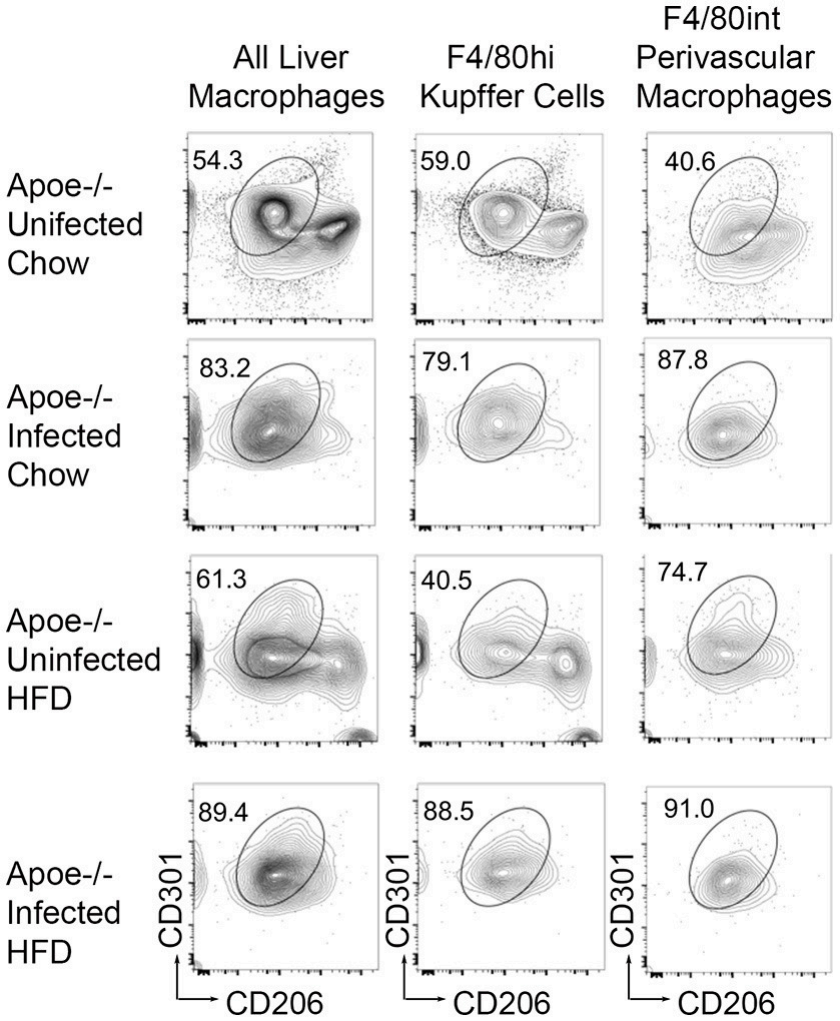
*S. mansoni* infection induces alternative macrophage activation even in the presence of high fat diet. ApoE^−/−^ male mice were fed HFD or standard chow diet for 10 days before infection with *S. mansoni*. At 10-weeks post infection, mice were sacrificed. Single cell suspension from perfused liver were stained with markers of interest and analyzed by flow cytometry using the macrophage gating strategy depicted in Figure 1. Data are concatenated from 5 to 9 mice per group and representative of four independent experiments.

**Figure 5:**
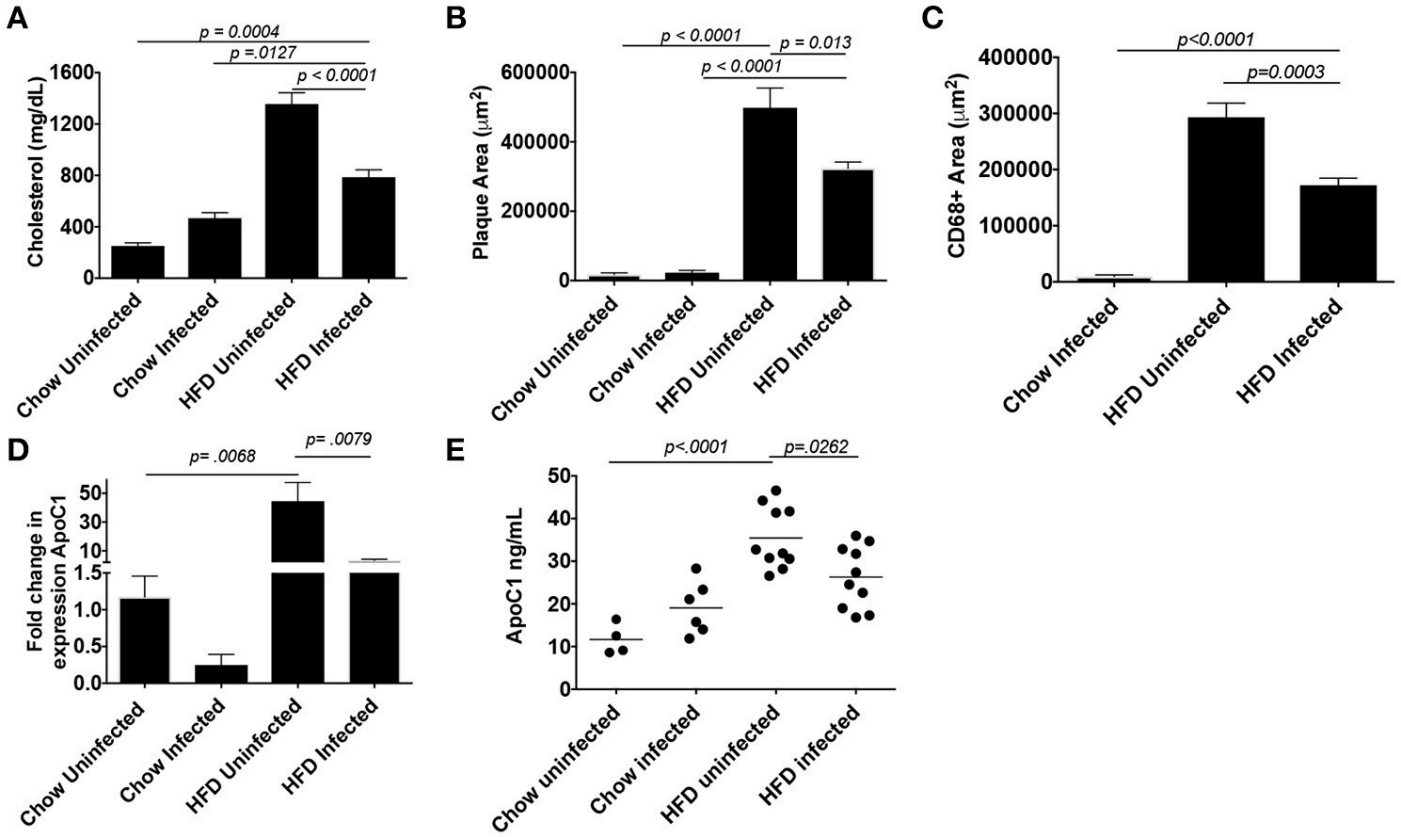
*S. mansoni* infection reduces hyperlipidemia, plaque, and macrophage area and well as systemic ApoC1 production in HFD fed ApoE^−/−^ mice. ApoE^−/−^ male mice were fed HFD or standard chow diet for 10 days before infection with *S. mansoni*. At 10-weeks post infection, mice were sacrificed. **(A)** Fasting plasma cholesterol measurements. **(B-C)** Quantitation of plaque and macrophage area (CD68^+^) from the aortic sinus. **(D)** QPCR analysis of *ApoC1* in whole liver RNA, data are normalized to Chow infected animals. **(E)** Quantitation of ApoC1 in plasma at the time of sacrifice. All data shown are two combined experiments with 4–8 mice per group in each experiment. The experiment was performed 4 times. Statistical significance was calculated using ANOVA and post-tests.

Schistosome infection in humans (26) and mice (15, 16, 27) protects from the development of obesity and atherosclerosis, but the molecular mechanism(s) that underlies this protection has yet to be fully uncovered. The transcriptional profile revealed by our analysis suggested that *S. mansoni*-induced metabolic alterations to hepatic macrophages could be involved in infection-induced protection from metabolic diseases. To assess this hypothesis, we employed the ApoE^−/−^ model of hyperlipidemia/atherosclerosis (28). ApoE^−/−^ mice were fed either a high-fat Western diet (HFD) or normal chow diet for 10 days before infection with *S. mansoni*, whereas controls were sham-infected. At 10-weeks post-infection we analyzed CD301 (galactose type C lectin) and CD206 (mannose receptor) expression as markers of alternative activation. Approximately 40–60% of macrophages from control chow uninfected were double positive, with the rest being CD206^+^ (Figure 4). Infection increased the frequency of alternative activation to ~80% regardless of diet, suggesting that HFD do not interfere with *S. mansoni* induced macrophage polarization. Similar to previous reports (15, 16), *S. mansoni*-infected mice on an HFD diet had significantly lower levels of plasma cholesterol and smaller aortic sinus plaques than uninfected HFD mice (Figures 5A,B). Plaques in the aortic sinus of HFD infected mice also contained fewer CD68^+^ macrophages (Figure 5C), suggesting reduced recruitment of monocytes to the plaque, as these macrophages have been previously shown to be monocyte-derived (29, 30). The transcriptional profile we uncovered in our analysis of schistosome-infected C57BL/6 liver macrophages (Figures 2, 3) showed reduced production of multiple apolipoproteins in macrophages, including ApoC1 (−10.00 log-FC, *p* = 1.35 × 10^−5^) and ApoC3 (−5.78 log-FC, *p* = 0.006). To confirm gene modulation on the ApoE^−/−^ model, we assessed the expression of a list of genes of interest. Importantly, ApoC1 was significantly downregulated in the same manner in both the C57BL/6 and ApoE^−/−^ model. Additionally, a previous report has indicated that ApoC1 production drives hyperlipidemia and pathogenesis in the ApoE^−/−^ model (31), making it a likely candidate for correlation with reduced plaque formation during infection. To address this possibility, we examined liver transcripts and plasma levels of ApoC1 and found that HFD induces an increase in ApoC1, but this increase is significantly attenuated by *S. mansoni* infection (Figures 5D,E). This reduction, in systemic ApoC1 production, is a component of the postulated mechanism for *S. mansoni*-related protection from HFD- induced atherosclerosis.

### Patent *S. mansoni* infection leads to improved glucose tolerance and alterations in hepatic macrophage amino acid biosynthesis and glucagon signaling pathways

**Figure 6:**
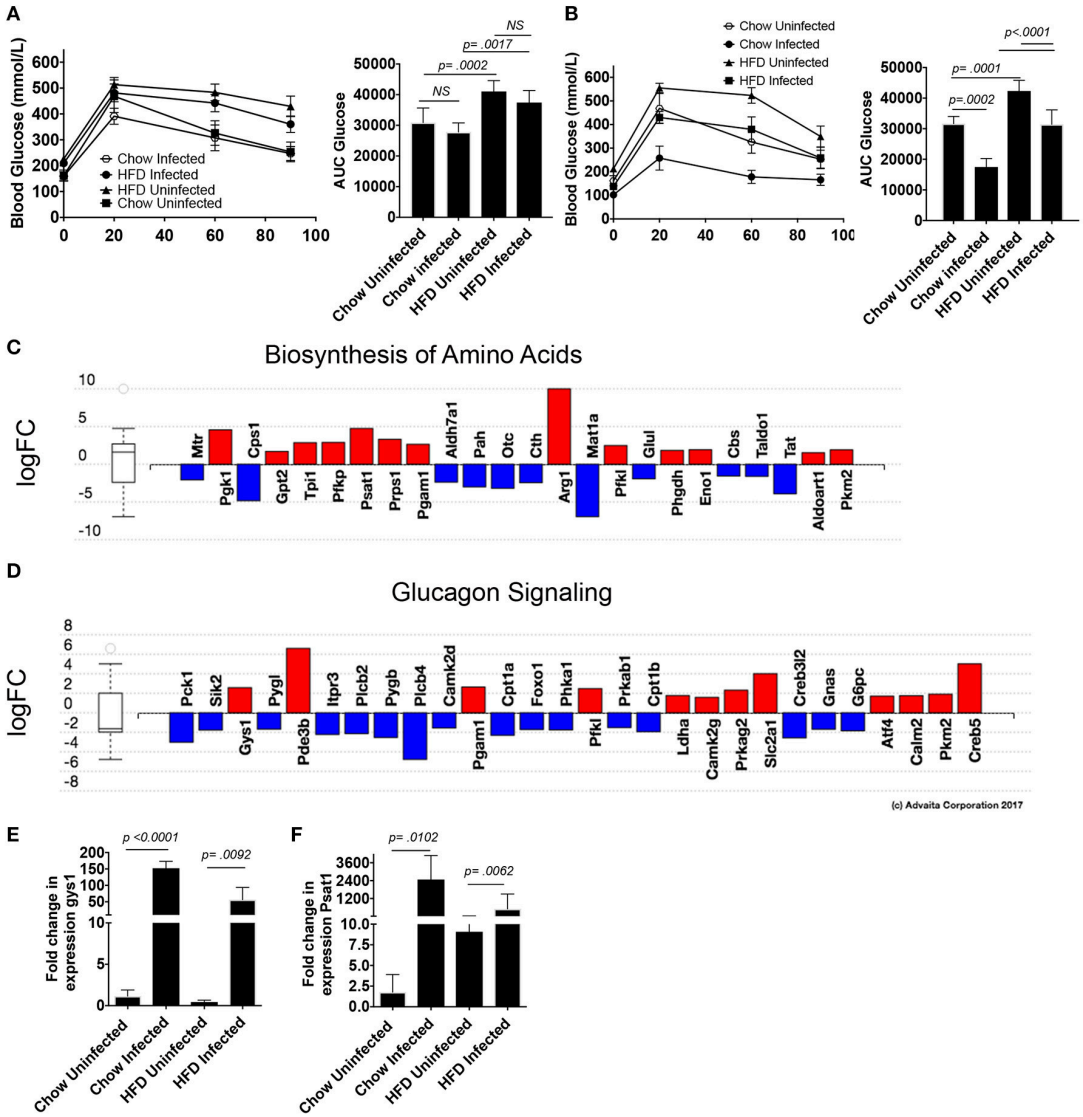
*S. mansoni* infection improves glucose tolerance and alters hepatic metabolism toward glycogen storage and amino acid biosynthesis. ApoE^−/−^ male mice were fed HFD or standard chow diet for 10 days before infection with *S. mansoni*. At 5 **(A)** and 10- **(B)** weeks post infection mice were fasted for 5 h and then administered an i.v GTT. **(C,D)** Perivascular macrophages and Kupffer cells were sorted from naïve and 10-week *S. mansoni*-infected C57BL/6 mice, RNA was extracted following Immgen protocols and populations were analyzed using whole-mouse genome Affymetrix Mouse Gene 2.0 ST Arrays. Significantly up (red) and down (blue) -regulated pathways were identified using Advaita Corporation iPathwayGuide. **(E,F)** QPCR analysis of gys1 and psat1 in whole liver RNA, data are normalized to Chow infected animals. Data in **(A,B,E,F)** are two combined experiments with 4-8 mice per group in each experiment and the experiment was performed 4 times. Statistical significance was calculated using ANOVA and post-tests.

Schistosomiasis has been reported to correlate with lower hemoglobin A1c levels in populations with a previous history of infection (26), as well in a murine model of obesity (27), so we sought to examine glucose tolerance in this model. *S. mansoni* induced reductions in serum cholesterol have previously been shown to be egg dependent, so we assessed glucose sensitivity in a 5-h glucose tolerance test (GTT) at 5-weeks post infection (pre-egg laying). We found that pre-patency, *S. mansoni* infection has no significant effect on glucose tolerance in normal chow fed mice as measured by area under the glucose excursion curve (AUC, Figure 6A). HFD increases blood glucose levels and decreases glucose tolerance (Figure 6A), while pre-patent *S. mansoni* infection has no significant effect on this increase. At the transition to chronic infection (10 weeks post infection), normal chow fed infected mice have lower fasting glucose levels and significantly improved whole-body glucose tolerance (Figure 6B). HFD significantly impairs glucose tolerance (as compared to chow uninfected controls), while *S. mansoni* infection of HFD fed mice restores glucose tolerance to a similar level as chow uninfected mice (Figure 6B). This is distinct from what has previously been reported, where *S. mansoni* infection of C57BL/6 mice improved glucose tolerance in HFD fed mice, but not normal low-fat chow fed mice. This difference suggests that there may be strain-specific differences in the control of glucose sensitivity. Since ApoE^−/−^ are prone to develop hyperlipidemia and atherosclerosis spontaneously as they age, it is possible that this propensity underlies the difference.

We then went back to the microarray pathway analysis and looked for pathway changes that could be expected to lead to improvement in glucose tolerance based on published reports. We found that *S. mansoni* infection significantly alters the biosynthesis of amino acids in Kupffer cells (Figure 6C, pathway analysis Bonferroni corrected *p* = 0.006), and one of the genes in that pathway, Psat1, stood out as a candidate for involvement in improving glucose tolerance as its transcript level is increased in infected C57BL/6 mice (logFC = 5,). The correlation between modulation of this transcript and infection was confirmed in our ApoE^−/−^ model (Figure 6C). Hepatic psat1 transcript levels (measured in total liver RNA) have previously been shown to be reduced in both genetic and diet-induced models of diabetes, while global overexpression of PSAT1 improves insulin signaling and sensitivity (19). The glucagon signaling pathway is also significantly altered (Figure 6D, pathway Bonferroni corrected *p* = 0.015), in that pathway, *S. mansoni* infection decreases pygl expression while also increasing gys1 transcripts. Genetic disruption of PYGL has previously been shown to improve glucose tolerance *in vivo* (32). While GYS1 is one of two enzymes responsible for glycogen synthesis, and small molecule induced activation of GYS1 in diabetic mice improves glucose tolerance (33). The combined effects of these two alterations would be predicted to lead to increased glucose tolerance and insulin sensitivity. We then asked whether gys1 transcripts are similarly altered by *S. mansoni* infection of HFD fed ApoE^−/−^ mice. *S. mansoni* infection leads to significantly higher gys1 transcripts in the liver tissue of both chow and HFD fed mice (Figure 6E). Psat1 is similarly altered by infection (Figure 6F), with transcripts significantly higher in *S. mansoni* infected mice on both chow and HFD, although transcript levels of HFD infected mice are lower than those of chow infected. These data suggest that the *S. mansoni* induced transcriptional changes that were identified via the microarray also occur in the liver tissue of HFD fed ApoE^−/−^ mice, supporting the hypothesis that *S. mansoni* induced alterations in hepatic macrophage metabolism are able to influence whole body metabolism and help protect from pathological changes generally associated with HFD induced metabolic disorders.

## Discussion

Overall, our data provide the first complete transcriptional analysis of Kupffer cells and perivascular macrophages in steady state and chronic *S. mansoni* infection. These data suggest that during *S. mansoni* infection, egg-induced M2 macrophages demonstrate profound alterations to their metabolic profile, with shifts in the balance of phospholipid and glucose metabolism as well as increased amino acid metabolism, and that these changes correlate with improvements in whole body cholesterol and glucose metabolism. While many of these alterations are shared between both perivascular macrophages and Kupffer cells, there are key genes that are differentially regulated in the two populations. Cbr2, Alox5, and F13a1 are all significantly up-regulated in Kupffer cells, but not in perivascular macrophages. Since Cbr2 and Alox are involved in arachidonic acid metabolism, it suggests that while both populations are alternatively activated, Kupffer cells specifically increase leukotriene production in the granuloma. Similarly, F13a1 has been previously implicated in wound healing (34), suggesting the Kupffer cells are more involved in the process of granuloma formation and resolution. Conversely, Lox is upregulated in both macrophage populations, but the increase in perivascular macrophages is far higher (8.3- vs. 2.1-fold), suggesting that perivascular macrophages are responding to greater oxidative stress during *S. mansoni* infection, a proposition that is supported by their tissue location. Many of the key markers of M2 activation (Chi3l3, Retnal, Mgl2) have a lower fold upregulation in perivascular macrophages, a finding that is matched by the frequency of these macrophages that are CD206^+^CD301^+^, suggesting that there may be degrees of alternative activation in the liver in the context of *S. mansoni* infection. The fact that only five genes (Subsets 1 and 2) are truly differentially regulated (opposite signs of significant fold changes) supports the hypothesis that after infection Kupffer cells and Perivascular macrophages act to a large degree in unison.

Recent work, using other models of M2 macrophage generation, have indicated that M2 macrophages rely on lipolysis driven fatty acid oxidation (35, 36) for the generation and maintenance of the M2 state. Our transcriptional analysis of Kupffer cells and perivascular macrophages suggest similar alterations are induced by chronic *S. mansoni* infection, but our data also provide the first evidence that alterations to hepatic macrophage metabolism either reflect or influence infection-induced alterations to whole body metabolism. Macrophages are known to be critical sources of apolipoproteins like ApoC1 (31, 37), and LPS-induced inflammation has been linked with increased levels of plasma cholesterol and ApoC1 (37). Our data indicating that *S. mansoni*-induced M2 transcriptional changes reduce both ApoC1 production and plasma cholesterol is in concordance with the established paradigm of M1 and M2 polarization states as driving opposite pathological states, and with the hypothesis that M2 macrophages play a role in the resolution and modulation of atherosclerotic lesions (38–40).

The liver plays a critical role in whole body metabolism through its control of glycogen metabolism, with glycogen synthase (GYS1) and glycogen phosphorylase (PYGL) acting to respectively increase and decrease stored glycogen to maintain blood sugar homeostasis. Our pathway analysis revealed key alterations to both macrophage amino acid and glucagon signaling/metabolic pathways. We identified key genes (GYS1, PYGL, and PSAT1) from both pathways that have previously been demonstrated to play a role in glucose tolerance/ insulin sensitivity in models of obesity and diabetes (19, 41) and epidemiological studies (42–44). We hypothesize that the combined effects of *S. mansoni*-induced alterations to these three genes are at least partially responsible for infection-related improvements in glucose tolerance in both chow and HFD fed mice, a proposition that will be tested in future studies. These data raise the possibility that *S. mansoni*-induced polarization of liver macrophages can significantly alter whole body metabolism. While multiple previous studies in humans (26, 45, 46) and mice (15, 16, 27) have indicated that helminth infections in general, and schistosomes in specific, modulate susceptibility to the development of metabolic diseases, a molecular mechanism underlying this modulation has not been identified. We postulate that *S. mansoni*-induced M2 polarization of hepatic macrophages are involved in protection from HFD induced hyperlipidemia, atherosclerosis, and glucose intolerance. Future studies will focus on thoroughly understanding the infection-induced transcriptional changes to other tissue macrophage populations and the broader monocyte lineage.

## Author contributions

KF, EP, and GR conceived of the project. KF, AR, DC-S, and GR planned the experiments. DC-S, AE, AR, and KF performed the experiments. KF, BR, DC-S, and AE analyzed the data. DC-S, KF, and BR wrote the manuscript.

### Conflict of interest statement

The authors declare that the research was conducted in the absence of any commercial or financial relationships that could be construed as a potential conflict of interest.
