# Supplementary figures and images for: Schistosoma mansoni Infection-Induced Transcriptional Changes in Hepatic Macrophage Metabolism Correlate With an Athero-Protective Phenotype
Source: Front Immunol. 2018 Nov 12;9:2580. doi: 10.3389/fimmu.2018.02580 (PMC6240656; doi:10.3389/fimmu.2018.02580)

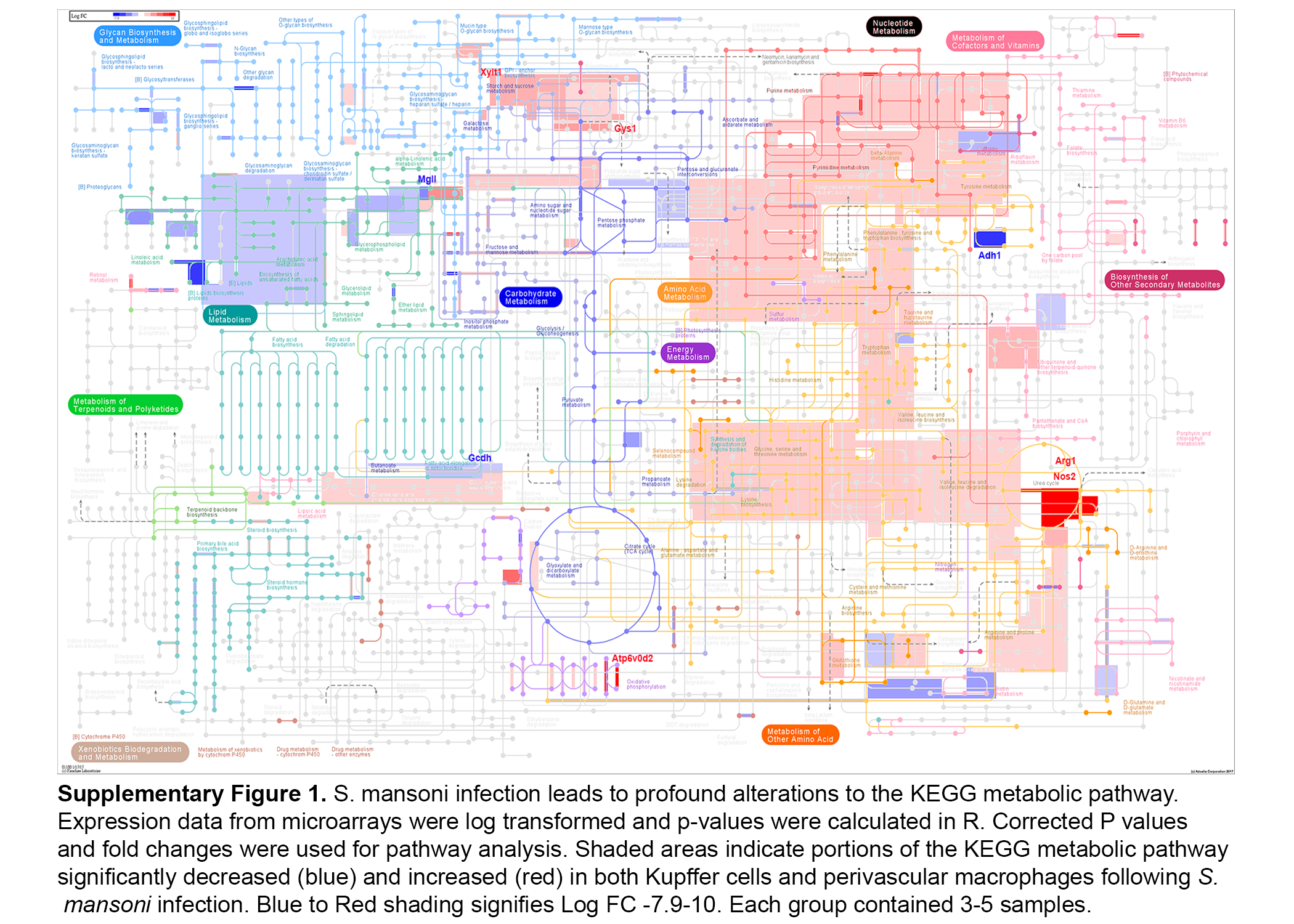

Supplement: Supplementary file 1 [file Image_1.TIF]
